# COVID-19 Higher Mortality in Chinese Regions With Chronic Exposure to Lower Air Quality

**DOI:** 10.3389/fpubh.2020.597753

**Published:** 2021-01-22

**Authors:** Riccardo Pansini, Davide Fornacca

**Affiliations:** ^1^Institute of Eastern-Himalaya Biodiversity Research, Dali University, Dali, China; ^2^Behavioral and Experimental Economics Research Center, Statistic and Mathematics College, Yunnan University of Finance and Economics, Kunming, China; ^3^Institute for Environmental Sciences, University of Geneva, Geneva, Switzerland

**Keywords:** air pollution, SARS-CoV-2, risk factors, virulence, climate change

## Abstract

We investigated the geographical character of the COVID-19 infection in China and correlated it with satellite- and ground-based measurements of air quality. Controlling for population density, we found more viral infections in those prefectures (U.S. county equivalent) afflicted by high Carbon Monoxide, Formaldehyde, PM 2.5, and Nitrogen Dioxide values. Higher mortality was also correlated with relatively poor air quality. When summarizing the results at a greater administrative level, we found that the 10 provinces (U.S. state equivalent) with the highest rate of mortality by COVID-19, were often the most polluted but not the most densely populated. Air pollution appears to be a risk factor for the incidence of this disease, despite the conventionally apprehended influence of human mobility on disease dynamics from the site of first appearance, Wuhan. The raw correlations reported here should be interpreted in a broader context, accounting for the growing evidence reported by several other studies. These findings warn communities and policymakers on the implications of long-term air pollution exposure as an ecological, multi-scale public health issue.

## Highlights

- There is a significant correlation between air pollution and COVID-19 spread and mortality in China.
- The correlation stands at a second-order administration level for several air pollutants, after controlling for varying population densities and removing Wuhan and Hubei from the dataset.
- Living in an area with low air quality is a risk factor for becoming infected and dying from this new form of coronavirus.

## Introduction

COVID-19, initially detected in China and rapidly spread to the rest of the world, has ignited a pandemic causing exorbitant human and economic cost (1). Within a few months since its discovery in December 2019, eastern and western doctors, biologists, and sociologists alike have turned their attention to disentangling the etiology of this airborne disease, a highly contagious respiratory illness caused by a novel coronavirus (2). Various risk factors have been implicated with the fast spread of the virus, assuming different characters, whether considered within or between countries. Even if free health care was dispensed to everyone in the exceptional case of this epidemics, the Chinese health system, like those of many other countries, is not adequate without proper identification and evaluation of the multiple epidemiological risk factors (3). On an individual level, an older age, the male gender and smoking status have all been shown to increase the coronavirus, SARS-CoV-2 (4). In particular, the angiotensin-converting enzyme 2 (ACE2) receptors in our respiratory system, hit by smoking and this new coronavirus, can bind with air pollutants (5).

From the standpoint of the natural sciences and geography, we can take a broader perspective to appreciate how a coronavirus, transmitted once more from an animal species to us, may show certain patterns in the way it affects and spreads among people, which go beyond virological and medical mechanisms, spatial proximity or apparently chaotic patterns. For example, elements including human and livestock overpopulation, biodiversity loss and climate change played a critical role in making the ground suitable for yet again a new epidemics to flourish (6). A multidisciplinary approach to study cultural and socioeconomic factors may be included when studying the likeliness of the populations to show stronger morbidity to this disease (7). Pertaining to climate change, air pollution is notoriously known to cause health problems and, in particular, viral respiratory infections and pneumonia to individuals chronically exposed to air pollutants (8, 9).

We therefore hypothesize a numerical and geographical association between chronic exposure to air pollution and the spread of SARS-CoV-2 (10). We investigated this possible correlation taking China as a unique case study (11), and have updated and expanded these findings here. A positive correlation had been found between chronic high levels of air pollution perceived as particulate matter found in 9 large Asian cities (three of those being Chinese) and higher lethality related to COVID-19 (12). Despite the strong containment measures adopted over there, if pollution still plays a role, it should be considered as an element of high concern in relation to this disease.

## Data and Methods

We collected COVID-19 infection and fatality figures for every prefecture of the People's Republic of China (2nd administrative divisions, equivalent to U.S. counties) from the Chinese government health commission (Table 1). We normalized these epidemiological values per 10,000 inhabitants of each prefecture. The data included COVID-19 cases and deaths.

**Table 1:** The analyzed datasets and their sources.

**Data**	**Measuring unit**	**Time period**	**Format**	**Source**
COVID-19	No. of infections, No. of deaths	Updated on 23 May 2020	Tabular prefecture level	DXY - DX Doctor: http://ncov.dxy.cn/ncovh5/view/en_pneumonia from Chinese government health commission
Population	No. of residents	Estimates 2017	Tabular prefecture level	https://www.citypopulation.de/ Data from provincial governments
**AIR QUALITY, GROUND STATIONS**				
PM2.5, PM10, O_3_, NO_2_, SO_2_, CO	Air Quality Index (AQI)	2014-2016	Tabular GPS points	University of Harvard Dataverse: https://dataverse.harvard.edu Data from http://aqicn.org
**AIR QUALITY, SATELLITE**				
UV Aerosol Index	Qualitative Index	2019	Continuous grid (0.01 arc deg.)	Sentinel-5 Atmospheric variables https://developers.google.com/earth-engine/datasets/tags/air-quality
CO, HCHO, NO_2_, O_3_, SO_2_	mol/m^2^			

The dataset of COVID-19 cases and deaths analyzed in this study captured the first and unique wave of SARS-CoV-2 infection for this country (19 December 2019–23 May 2020). It includes the 17 April update, when an increase of 1,290 casualties was reported, following a revised WHO guideline, showing a drastic rise of about 50% from the prior figure. This update included an increase of 325 infections for the city of Wuhan only.

While infections and fatalities inform on the extent of the pandemic, mortality rates (fatalities/infections ^*^ 100) provide additional information on the severity of the virus in each prefecture. It is important to note that China did not see a systematic COVID-19 testing at a national scale. Tests were mostly performed for people presenting symptoms and registered in hospitals. In some later cases, large scale testing was performed only to prevent localized outbreaks. As a result, asymptomatic cases are not included in the data, and mortality rates may appear inflated compared to other regions of the world.

Population densities were defined using the population totals of each prefecture divided by its surface area. Air pollution measurements from localized ground stations (monthly averages 2014–2016) as well as continuous tropospheric vertical column density measurements (whole year 2019) of several air pollutants were aggregated as the average values at the prefecture level. Time series information of atmospheric air pollutants was retrieved by the Sentinel-5, a satellite mission launched in October 2017 as part of the Copernicus program of the European Space Agency (13). The Google Earth Engine platform (14) was employed to compute the 2019 averages of each air pollutant measurements derived from satellite, namely the UV Aerosol Index, Carbon Monoxide (CO), Formaldehyde (HCHO), Nitrogen Dioxide (NO_2_), Ozone (O_3_), and Sulfur Dioxide (SO_2_). Air pollutants collected from ground stations were PM 2.5, PM 10, O_3_, NO_2_, SO_2_, and CO. Data types and their sources are shown in Table 1 and the fully compiled dataset, including an aggregated version at the provincial level, are available on a dedicated GitHub repository (https://github.com/DavideFornacca/COVID19/tree/master/China).

Correlation and significance analyses between air pollution, population, and the three COVID-19 variables (infections/100,000 inhabitants, fatalities/100,000 inhabitants, mortality rate) were performed for the prefecture-level dataset using non-parametric Kendall rank correlation coefficient because of the distributions of COVID-19 and population variables being mostly skewed. To assess the potential influence of outliers, we repeated the same tests by firstly removing the prefecture of Wuhan and then the whole Hubei province from the dataset. The significance threshold was set to <0.05. Using the aggregated version of the dataset (mean values at the provincial level), we identified the first 10 Chinese provinces showing the highest values of each variable separately, and we used this for comparative analysis. Furthermore, thematic maps comparing COVID-19 and air pollution distributions in China were produced for visual assessment.

Data processing and mapping was done with QGIS. Statistical analysis was performed in Python programming environment.

## Results

A descriptive statistics' summatory table for the different satellite- and ground-based air quality measurements can be found in Supplementary Table 1.

Higher amounts of viral infections per 100,000 inhabitants, fatalities per 100,000 inhabitants, and mortality rates (fatalities/infections ^*^ 100) were found in those Chinese prefectures afflicted by several pollutants of the air: CO, HCHO, PM 2.5, PM 10, and NO_2_, as shown by the significant positive correlation coefficients in Table 2. In particular, stronger associations for infections and fatalities were found with tropospheric column values of Formaldehyde (*r*_τ_ = 0.34, *p* < 0.001 and *r*_τ_ = 0.20, *p* < 0.001) and Carbon Monoxide values (*r*_τ_ = 0.28, *p* < 0.001 and *r*_τ_ = 0.20, *p* < 0.001), while for mortality rates, PM 2.5 was the most incident pollutant (*r*_τ_ = 0.18, *p* < 0.001). This trend holds also after removing in succession (i) Wuhan city and (ii) the whole Hubei province from the dataset (see Supplementary Tables 3, 4). Levels of particulate matter measured by ground stations, especially the finer PM 2.5, were associated with a greater number of fatalities and higher mortality rates. Conversely, aerosol data from the satellite, which potentially include PM 2.5 and PM 10, were not associated with fatalities or mortality rates. They negatively correlated with infections weakly. This is not surprising, given that the measurement is related to UV-absorbing particles, which are in general non-pollutant, being inert particulates such as dust, sand, and sea salt, but they also include smoke from volcano ash and biomass burning. To note that these sources of dust, however, are often found far from highly-polluted development areas. Higher levels of O_3_ and SO_2_ from both satellite and ground data were not associated with more COVID-19 deaths and mortality rates. This goes against the trend shown by the other pollutants, an aspect that will require further investigation. Levels of CO, HCHO, and PM 2.5 showed stronger correlation than the population variables when analyzing fatalities and mortality rates. As expected, several air pollutants correlated with population density except for Sulfur Dioxide. Conversely, O_3_ and Aerosol index showed weak negative correlations, suggesting their presence in low populated areas (Supplementary Table 2). A comprehensive statistical output of these data is reported in Table 2.

**Table 2:** Correlation between satellite- and ground-based air quality variables with (i) cumulated COVID-19 infections per 100,000 inhabitants, (ii) fatalities per 100,000 inhabitants, and (iii) mortality rate (fatalities / infections) in China at a prefectural level, until 23 May 2020.

	**Infections**			**Fatalities**			**Mortality**		
	**(/100k pop)**			**(/100k pop)**			**(fatalities/infections)**		
	**df (n-2)**	**tau**	***p*-value**	**df (n-2)**	**tau**	***p*-value**	**df (n-2)**	**tau**	***p*-value**
**CO sat**	337	0.28	** <0.001**	337	0.19	** <0.001**	313	0.16	** <0.001**
**NO**_**2**_ **sat**	337	0.23	** <0.001**	337	0.14	**0.001**	313	0.12	**0.006**
**O**_**3**_ **sat**	337	−0.08	**0.030**	337	0.00	0.967	313	0.02	0.635
**SO**_**2**_ **sat**	337	−0.10	**0.005**	337	−0.02	0.634	313	0.00	0.964
**Aerosol sat**	337	−0.12	**0.001**	337	−0.03	0.488	313	0.00	0.950
**HCHO sat**	337	0.34	** <0.001**	337	0.20	** <0.001**	313	0.17	** <0.001**
**PM 2.5 ground**	302	0.15	** <0.001**	302	0.18	** <0.001**	285	0.18	** <0.001**
**PM 10 ground**	302	0.04	0.330	302	0.12	**0.006**	285	0.13	**0.005**
**CO ground**	302	−0.01	0.840	302	0.11	**0.012**	285	0.12	**0.007**
**NO**_**2**_ **ground**	302	0.12	**0.002**	302	0.12	**0.005**	285	0.12	**0.007**
**O**_**3**_ **ground**	302	−0.03	0.477	302	−0.02	0.585	285	−0.03	0.482
**SO**_**2**_ **ground**	302	−0.01	0.843	302	0.04	0.409	285	0.06	0.178
**population**	337	0.23	** <0.001**	337	0.17	** <0.001**	313	0.14	** <0.001**
**pop density**	337	0.32	** <0.001**	337	0.16	** <0.001**	313	0.12	**0.004**

*Note that the degrees of freedom (df) are different for the ground station results because of the limited data availability. p < 0.05 are marked in bold characters*.

The values shown in Table 3 include the prefectural detail aggregated at a coarser provincial level, and they are sorted to highlight the 12 provinces with the highest rates of mortality. These provinces are often reported among the 12 most polluted ones, except for Taiwan and Hainan. However, these provinces are not the most densely populated, with the exception of Henan, Taiwan (a densely populated island), Tianjin and Beijing (two relatively small but highly populated provincial-level municipalities).

**Table 3:** Summary table of mean values of COVID-19 (orange), air pollution (blue), and population density (green) variables at the provincial level.

**Province (prefectures *n*)**	**Pop density (pop/m^**2**^)**	**Total infections**	**Infections/100k pop**	**Total fatalities**	**Fatalities/100k pop**	**Mortality (%)**	**CO sat (μmol/m^**2**^)**	**NO_**2**_ sat (μmol/m^**2**^)**	**O_**3**_ sat (μmol/m^**2**^)**	**SO_**2**_ sat (μmol/m^**2**^)**	**Aerosol sat (index)**	**HCHO sat (μmol/m^**2**^)**	**PM25 gr (AQI)**	**PM10 gr (AQI)**	**CO gr (AQI)**	**NO_**2**_ gr (AQI)**	**O_**3**_ gr (AQI)**	**SO_**2**_ gr (AQI)**
Hubei (15)	378.92	68,135	115.44 ± 106.39	4,512	7.6449 ± 8.3312	6.62 ± 1.63	46,393 ± 4845	51.60 ± 21.80	123,670 ± 1,342	20.17 ± 13.46	−1.04 ± 0.03	176.50 ± 27.82	133 ± 13	71 ± 12	10 ± 3	13 ± 4	24 ± 5	11 ± 3
Hainan (3)	541.16	169	1.83 ± 3.25	6	0.0648 ± 0.0660	3.55 ± 3.44	40,725 ± 1,175	21.00 ± 4.42	116,021 ± 101	−19.02 ± 8.73	−1.11 ± 0.09	146.33 ± 17.63	61 ± 5	29 ± 4	6 ± 0	6 ± 1	20 ± 2	2 ± 1
Heilongjiang (13)	93.29	559	1.47 ± 0.98	13	0.0341 ± 0.0580	2.33 ± 2.76	38,689 ± 1,588	21.34 ± 8.40	165,885 ± 2,284	93.47 ± 37.60	−0.93 ± 0.08	94.29 ± 16.13	91 ± 20	49 ± 11	6 ± 2	10 ± 4	24 ± 5	9 ± 4
Gansu (14)	124.46	91	0.35 ± 0.34	2	0.0076 ± 0.0142	2.20 ± 1.68	28,325 ± 3,199	25.23 ± 13.34	132,895 ± 4,450	72.38 ± 29.55	−0.79 ± 0.20	91.62 ± 17.26	98 ± 13	69 ± 14	10 ± 3	14 ± 5	30 ± 9	13 ± 5
Jilin (9)	165.48	154	0.56 ± 0.20	3	0.0109 ± 0.0098	1.95 ± 2.36	40,300 ± 1,983	36.23 ± 14.48	158,741 ± 2,422	105.60 ± 11.59	−0.93 ± 0.15	104.95 ± 12.74	113 ± 19	64 ± 12	9 ± 2	13 ± 3	24 ± 2	12 ± 3
Hebei (11)	514.53	318	0.42 ± 0.25	6	0.0080 ± 0.0144	1.89 ± 3.40	51,139 ± 7,776	129.64 ± 46.39	144,916 ± 4,197	190.11 ± 46.96	−0.92 ± 0.08	175.63 ± 41.13	157 ± 30	108 ± 28	12 ± 3	21 ± 5	24 ± 3	23 ± 6
Xinjiang (16)	145.23	56	0.23 ± 0.23	1	0.0041 ± 0.0490	1.79 ± 9.45	28,671 ± 4,747	23.41 ± 20.33	142,304 ± 4,832	68.02 ± 74.28	−0.53 ± 0.32	78.33 ± 17.42	108 ± 32	87 ± 43	11 ± 3	13 ± 5	24 ± 5	7 ± 2
Henan (17)	675.57	1,273	1.33 ±0.87	22	0.0229 ± 0.0189	1.73 ± 3.40	49,781 ± 3,351	96.52 ± 30.76	131,876 ± 2,818	93.28 ± 38.83	−1.01 ± 0.03	190.87 ± 14.88	145 ± 8	91 ± 9	15 ± 4	19 ± 4	23 ± 6	21 ± 7
Taiwan (1)	648.32	441	1.87	7	0.0297	1.59	34,858	39.1	115,054	−21.2	−1.09	121.27	–	–	–	–	–	–
Liaoning (14)	319.55	128	0.29 ± 0.20	2	0.0046 ± 0.0133	1.56 ± 4.82	45,853 ± 2,591	72.03 ± 21.47	153,539 ± 2,061	154.65 ± 17.93	−0.97 ± 0.08	117.78 ± 8.57	120 ± 10	70 ± 8	13 ± 3	17 ± 3	25 ± 4	22 ± 5
Tianjin (1)	1330.81	192	1.23	3	0.0193	1.56	54,124	175.04	146,921	193.42	−0.87	198.66	135	84	14	21	21	17
Beijing (1)	1313.10	593	2.75	9	0.0418	1.52	45,974	123.19	148,350	134.26	−0.94	173.28	145	83	11	24	27	11
Guizhou (9)	244.30	146	0.41 ± 0.19	2	0.0056 ± 0.0125	1.37 ± 3.35	38,014 ± 1,975	30.87 ± 7.21	117,525 ± 910	21.63 ± 12.63	−0.96 ± 0.07	136.68 ± 5.70	106 ± 7	54 ± 7	7 ± 0	13 ± 1	20 ± 1	10 ± 1
Inner Mongolia (12)	73.21	77	0.30 ± 0.33	1	0.0039 ± 0.0171	1.30 ± 3.77	33,581 ± 2,486	33.47 ± 32.68	150,964 ± 8,343	92.09 ± 48.43	−0.78 ± 0.15	81.38 ± 13.90	90 ± 18	58 ± 18	9 ± 6	11 ± 6	27 ± 7	14 ± 10
Shaanxi (10)	280.14	243	0.63 ± 0.41	3	0.0078 ± 0.0106	1.23 ± 0.79	37,972 ± 3,157	51.47 ± 26.39	130,735 ± 4,029	69.58 ± 22.96	−0.97 ± 0.04	140.57 ± 20.20	121 ± 20	72 ± 16	15 ± 5	16 ± 4	20 ± 3	16 ± 11
Yunnan (18)	127.40	174	0.36 ± 0.33	2	0.0041 ± 0.0229	1.15 ± 2.51	31,788 ± 4,068	18.43 ± 6.32	115,465 ± 593	18.41 ± 15.22	−1.23 ± 0.08	118.73 ± 22.77	74 ± 12	37 ± 8	7 ± 2	7 ± 2	26 ± 6	8 ± 4
Shanghai (1)	3513.05	667	2.76	7	0.0289	1.05	45,641	178.15	124,858	54.4	−0.94	156.15	116	57	7	21	35	9
Chongqing (1)	376.25	579	1.87	6	0.0193	1.04	41,471	35.8	121,772	20	−0.93	150.42	124	62	9	19	18	10
Shandong (15)	641.28	763	0.76 ± 0.69	7	0.0070 ± 0.0140	0.92 ± 2.06	51,789 ± 2,492	125.14 ± 22.32	138,473 ± 2,630	152.21 ± 23.38	−1.00 ± 0.03	178.85 ± 21.53	146 ± 22	94 ± 18	11 ± 4	21 ± 4	31 ± 6	25 ± 8
Guangxi (14)	247.65	252	0.51 ± 0.76	2	0.0041 ± 0.0171	0.79 ± 1.13	43,533 ± 1,523	29.83 ± 4.08	116,588 ± 312	−16.53 ± 8.68	−0.94 ± 0.04	160.19 ± 8.65	97 ± 13	47 ± 7	9 ± 2	9 ± 3	26 ± 7	9 ± 3
Anhui (15)	673.01	991	1.57 ± 1.26	6	0.0095 ± 0.0359	0.61 ± 0.78	47,941 ± 1,974	80.50 ± 22.67	126,466 ± 2,866	56.81 ± 13.74	−1.08 ± 0.03	181.35 ± 7.91	117 ± 16	59 ± 10	8 ± 2	13 ± 3	25 ± 5	11 ± 4
Sichuan (19)	385.74	563	0.68 ± 1.36	3	0.0036 ± 0.0041	0.53 ± 0.40	38,603 ± 7,695	37.53 ± 16.58	121,486 ± 2,521	36.45 ± 23.37	−0.97 ± 0.12	144.81 ± 35.70	109 ± 27	57 ± 14	8 ± 2	13 ± 4	25 ± 6	9 ± 4
Guangdong (19)	1270.33	1,590	1.40 ± 1.39	8	0.0071 ± 0.0143	0.50 ± 2.42	42,819 ± 1,462	66.15 ± 43.52	116,397 ± 212	−32.14 ± 13.12	−1.05 ± 0.07	168.06 ± 23.56	93 ± 7	44 ± 4	9 ± 1	12 ± 4	28 ± 5	7 ± 2
Hunan (14)	356.49	1,018	1.48 ± 0.82	4	0.0058 ± 0.0082	0.39 ± 0.35	45,067 ± 2,212	37.94 ± 10.56	119,555 ± 1,383	1.25 ± 9.58	−0.99 ± 0.03	173.23 ± 17.23	114 ± 15	59 ± 8	9 ± 2	11 ± 4	27 ± 6	12 ± 3
Hong Kong SAR (1)	6598.01	1,065	14.29	4	0.0537	0.38	42,420	139.17	116,276	−48.4	−1.02	176.42	92	46	10	17	28	4
Fujian (9)	652.50	296	0.75 ± 0.49	1	0.0025 ± 0.0043	0.34 ± 0.46	39,331 ± 848	36.99 ± 12.96	117,021 ± 627	−10.92 ± 11.61	−1.09 ± 0.05	142.37 ± 10.48	76 ± 10	37 ± 7	7 ± 1	10 ± 3	24 ± 7	5 ± 2
Jiangxi (11)	340.40	934	2.02 ± 0.23	1	0.0022 ± 0.0035	0.11 ± 0.40	45,074 ± 1,956	43.39 ± 10.75	119,799 ± 1,251	18.69 ± 14.93	−1.07 ± 0.03	173.25 ± 11.72	100 ± 7	51 ± 7	7 ± 2	10 ± 3	21 ± 4	12 ± 4
Zhejiang (11)	641.36	1,182	2.09 ± 1.45	1	0.0018 ± 0.0033	0.08 ± 0.06	42,655 ± 2,191	71.76 ± 34.48	121,312 ± 1,686	20.05 ± 14.83	−1.05 ± 0.05	163.04 ± 19.40	114 ± 10	55 ± 6	8 ± 1	17 ± 4	30 ± 4	9 ± 3
Jiangsu (13)	856.03	631	0.78 ± 2.99	0	0	0	48,727 ± 1,420	125.84 ± 30.98	128,531 ± 2,714	75.05 ± 24.14	−1.05 ± 0.03	175.42 ± 13.30	120 ± 8	66 ± 8	5 ± 1	16 ± 4	29 ± 3	12 ± 3
Shanxi (11)	270.16	134	0.36 ± 0.27	0	0	0	39,921 ± 4,031	89.14 ± 17.85	138,704 ± 3,975	150.68 ± 27.36	−1.05 ± 0.05	136.37 ± 25.60	125 ± 11	77 ± 14	17 ± 4	17 ± 3	24 ± 5	28 ± 4
Ningxia (5)	155.56	74	1.09 ± 0.85	0	0	0	32,917 ± 3,598	49.00 ± 28.86	136,458 ± 3,274	121.81 ± 43.19	−0.82 ± 0.06	103.16 ± 9.05	112 ± 0	81 ± 1	9 ± 1	15 ± 3	24 ± 1	26 ± 5
Macao SAR (1)	19136.39	45	6.89	0	0	0	43,309	125.72	116,336	−42.5	−0.98	170.45	–	–	–	–	–	–
Qinghai (8)	60.22	18	0.30 ± 0.41	0	0	0	20,107 ± 3,157	13.54 ± 10.12	129,362 ± 3,269	36.08 ± 36.84	−0.89 ± 0.23	61.29 ± 12.75	109 ± 18	71 ± 5	10 ± 3	11 ± 6	31 ± 8	12 ± 3
Xizang (7)	5.70	1	0.03 ± 0.07	0	0	0	17,503 ± 1,374	8.91 ± 1.60	118,934 ± 3,000	6.94 ± 6.13	−0.86 ± 0.22	59.39 ± 5.16	62 ± 25	37 ± 23	8 ± 5	6 ± 2	26 ± 5	6 ± 5

*Colored cells represent the 12 highest values for each column. The table is in descending order according to mortality rates. Standard deviations of COVID-19 and pollution variables are also displayed. Sat, satellite measures; gr, ground stations; AQI, Air Quality Index*.

**Figure 1:**
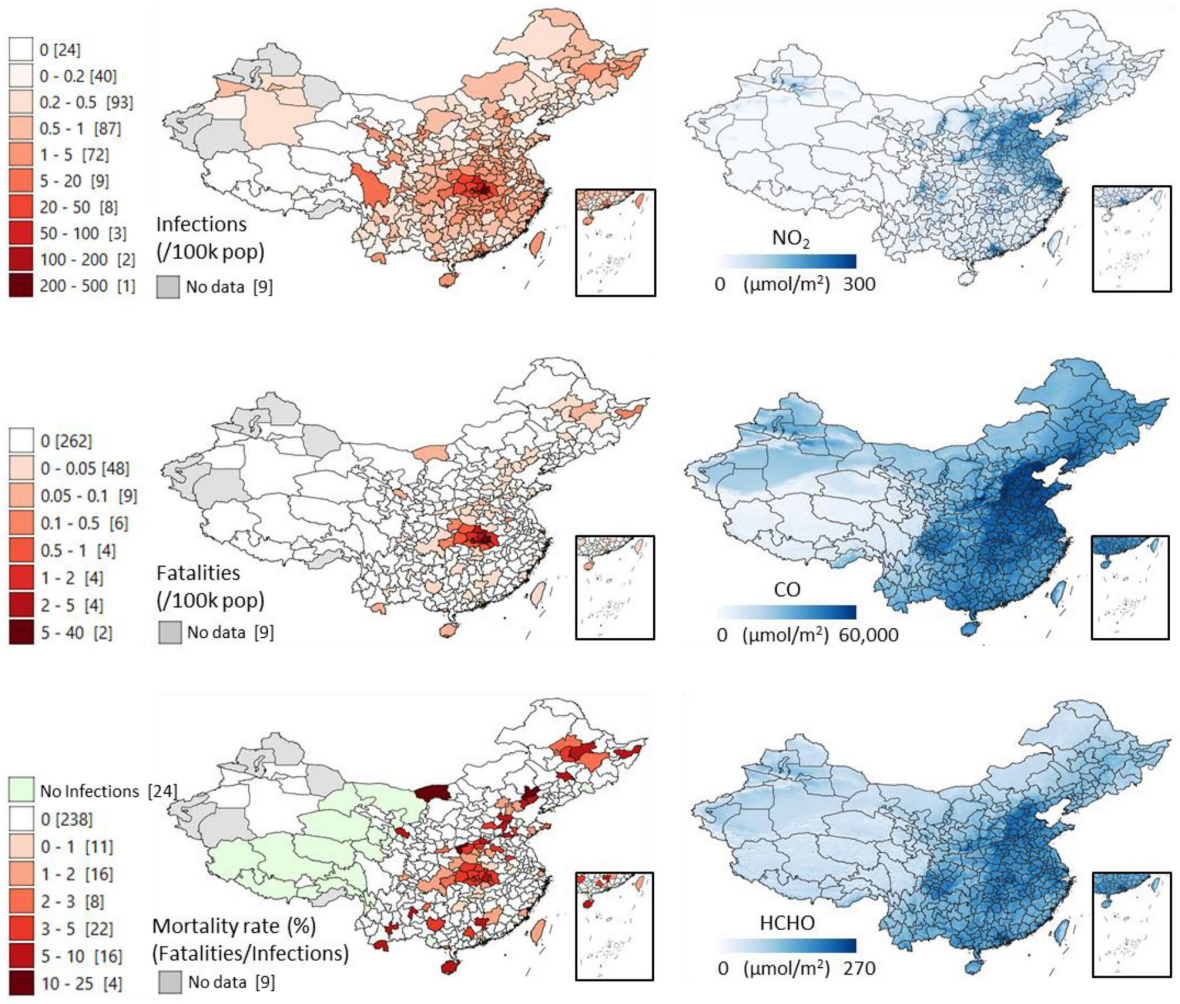
Distribution of COVID-19 infections, fatalities and mortality rates (fatalities/infections * 100) across the prefectures of China (updated on 23 May 2020), and the distribution of the tropospheric column amounts of three representative air pollutants derived from the 2019 averaged satellite measures of: Nitrogen Dioxide (NO_2_), Carbon Monoxide (CO), and Formaldehyde (HCHO). The values in the square brackets show the COVID-19 cases' counts of administrative units.

The maps (Figure 1) offer a synoptic view of the correlations. Those ones referring to different pollutants display continuous values at 0.01 arc-degree (about 1 km) resolutions, which can potentially highlight within-prefecture differences. Prefectural mean values of each air pollutant can be found in the dataset available in the dedicated repository. A clear longitudinal pattern ranging from the northeast region down to Hong Kong is visible for both COVID-19 variables and air pollution. In addition to Wuhan and Hubei appearing heavily affected compared to the rest of the country, the map shows mortality occurring in several other hotspots, also in less populated yet industrial provinces of central China.

## Discussion and Conclusion

The present study suggests a strong association between the incidence of COVID-19 and chronic exposure to air pollution in China. Comparative analyses made in this study indicate the role of air pollution as a critical risk cofactor for COVID-19 in China, with a stronger influence of Formaldehyde and Carbon Monoxide levels.

Our finding is in line with other studies observing a similar influence of air pollutants. (i) Firstly, testing the more proximal hypothesis that COVID-19 outbreaks could follow with a temporal delay from days with high NO_2_ presence in the air, colleagues in Shanghai have published detailed time series data pointing at a lag of 12 days before hospitalizations for the Hubei province (16). This suggests the role of air pollutants as airborne vectors for this virus, also highlighted by another study (18) conducted in three cities in Hubei province and a further one illustrating a potential role of PMs in other Asian cities (12). (ii) Secondly, in the United States, the correlation of respiratory illness with chronic exposure to PM 2.5 was observed stronger than 11 other demographic covariables, including population density, patients' age, socioeconomic status, ethnicity, education, obesity, smoking status, number of hospital beds per unit population, average daily temperature and relative humidity, and lockdown state (15) This same finding for the U.S. was replicated by others, e.g., (17). (iii) Thirdly, in Italy, a similar positive correlation between COVID-19 occurrence and chronic exposure to NO_2_, O_3_ and PMs was also reported (20) controlling in addition for the extra five demographic variables of mobility, temperature, housing density, health care density, and age of the population (21), with the additional and novel evidence that fragments of the RNA from this virus were found in the particulate matter of the harshly hit northern Italian city of Bergamo (19), laying in the most polluted European area of the Po valley, severely affected by the virus (22). (iv) Finally, our couple of multinational investigations employing different satellite-based datasets has also observed trends similar to the current paper in at least five countries other than China, including Italy, United States, Iran, France, and U.K. (23, 24). Having run and posted these significant correlations at different moments throughout the expansion of the pandemic is an element which further signals that the correlations stand throughout time.

Our study combined data from two different sources: localized ground-stations, directly measuring ambient air pollution, and continuous, grid-based satellite observations. These two sources present several technical and methodological differences that could explain some degree of discordance in the resulting correlation analyses, such as the one found between the satellite-based UV Aerosol Index and the ground values of particulate matter. We maintained the assessment of pollutants from the traditional ground stations because they are more representative of pollution levels to which populations living in their proximity are exposed. Notwithstanding that, they are unevenly scattered and their spatial coverage is very limited. On the other hand, satellite observations offer the unique advantage of global coverage, highlighting pollution differences at a regular and finer spatial unit. We believe that the combined use of these two data sources is instrumental in interpreting any detrimental role of specific air pollutants.

A significant obstacle to the interpretability of these findings in China is the availability of only limited information about the associated covariables, such as high-resolution data regarding health services and infrastructures, epidemiological traits, and population movement, to ascertain the relative importance of air pollution among many socio-environmental driving factors of COVID-19 infections. For example, the spatial effect of the strict lockdown adopted in China can be deduced by the huge difference in the number of infections and fatalities between Wuhan, its surrounding prefectures, and the rest of China (see maps in Figure 1). We have considered population density as chief cofactor and found that its correlation with COVID-19 is similar to air pollution, although slightly weaker. Moreover, highly populated areas are often more polluted (Supplementary Table 2). These results preclude us from understanding specific health consequences of air pollutants and call for a pressing need to further investigate this matter. In the past, the correlation between air pollution and human illness was notified and attributable to PMs and NO_2_ acting as vectors for the spread and extended survival of bioaerosols (25–30) in relation to pathogenic microbes including the avian influenza, measles and the syncytial virus (31–35). To generalize the results of the present study, higher mortality rates were found in provinces with the worst air pollution problems and only some of them were among the most densely populated ones. Regardless, the probability of dying from the virus once it infects is higher where air pollution is heavier. We have also observed a similar pattern for populations at risk of chronic exposure of PM 2.5 and NO_2_ in the two less densely-populated countries of Italy and Iran (23).

To conclude, despite the fact the SARS-CoV-2 was first detected in Wuhan and that the first location of the pathogen assumes a key role in the geographical spread of the infection, the detrimental effect of air pollution on patients infected by the virus remains evident. To overcome the limitations of our study, longitudinal screenings performed on patients from retrospective cohorts will help clarify the role of air pollution as a cofactor for these types of airborne transmittable diseases (36). In this century, in China as elsewhere, health policymaking is not adequate unless following human and environmental “one health” approaches. As a clear and immediate action to prevent the trajectory of this and future epidemics, curbing climate change (37) must be endorsed way more seriously. Will the smallest of the parasites be able to awaken us, this time, so that we convincingly start caring about the health of the environment as much as we have clumsily been caring about our public health?

## Data Availability Statement

The data are available at https://github.com/DavideFornacca/COVID19/tree/master/China.

## Author Contributions

RP: concept. RP and DF: design, interpretation, drafting of the manuscript, and critical revision of the manuscript. DF: data acquisition and statistical analysis. Both authors contributed to the article and approved the submitted version.

## Conflict of Interest

The authors declare that the research was conducted in the absence of any commercial or financial relationships that could be construed as a potential conflict of interest.

## References

[1] Bashir MF Ma B Shahzad L. A brief review of socio-economic and environmental impact of Covid-19. Air Qual Atmos Health. (2020) 13:1403–9. 10.1007/s11869-020-00894-8 32837620 PMC7395801

[2] Wang C Horby PW Hayden FG Gao GF. A novel coronavirus outbreak of global health concern. Lancet. (2020) 395:470–3. 10.1016/S0140-6736(20)30185-9 31986257 PMC7135038

[3] Zhai T Goss J. Health system reform in China: the challenges of multimorbidity. Lancet Global Health. (2020) 8:e750–1. 10.1016/S2214-109X(20)30225-4 32446340

[4] Zhou F Yu T Du R Fan G Liu Y Liu Z Clinical course and risk factors for mortality of adult inpatients with COVID-19 in Wuhan, China: a retrospective cohort study. Lancet. (2020) 395:1054–62. 10.1016/S0140-6736(20)30566-3 32171076 PMC7270627

[5] Lukassen S Chua RL Trefzer T Kahn NC Schneider MA Muley T. SARS-CoV-2 receptor ACE2 and TMPRSS2 are primarily expressed in bronchial transient secretory cells. EMBO J. (2020) 39:e105114. 10.15252/embj.2020105114 32246845 PMC7232010

[6] Bedford J Farrar J Ihekweazu C Kang G Koopmans M Nkengasong J. A new twenty-first century science for effective epidemic response. Nature. (2019) 575:130–6. 10.1038/s41586-019-1717-y 31695207 PMC7095334

[7] Bontempi E Vergalli S Squazzoni F. Understanding COVID-19 diffusion requires an interdisciplinary, multi-dimensional approach. Environ Res. (2020) 188:109814. 10.1016/j.envres.2020.109814 32544726 PMC7289085

[8] Lelieveld J Evans JS Fnais M Giannadaki D Pozzer A. The contribution of outdoor air pollution sources to premature mortality on a global scale. Nature. (2015) 525:367–71. 10.1038/nature15371 26381985

[9] Krewski D. Evaluating the effects of ambient air pollution on life expectancy. N Engl J Med. (2009) 360:413–5. 10.1056/NEJMe0809178 19164194

[10] Comunian S Dongo D Milani C Palestini P. Air pollution and Covid-19: the role of particulate matter in the spread and increase of Covid-19's morbidity and mortality. Int J Environ Res Public Health. (2020) 17:4487. 10.3390/ijerph17124487 32580440 PMC7345938

[11] Pansini R Fornacca D COVID-19 higher morbidity and mortality in Chinese regions with lower air quality. medRxiv. (2020). 10.1101/2020.05.28.20115832 PMC7874038 33585383

[12] Gupta A Bherwani H Gautam S Anjum S Musugu K Kumar N. Air pollution aggravating COVID-19 lethality? Exploration in Asian cities using statistical models. Environ Dev Sustain. (2020) 1–10. 10.1007/s10668-020-00878-9 32837279 PMC7362608

[13] ESA European Space Agency - Sentinel-5. (2018). Available online at: https://developers.google.com/earth-engine/datasets/tags/air-quality (accessed March 25, 2020).

[14] Google Google Earth Engine. (2020). Available online at: https://developers.google.com/earth-engine/datasets/catalog/sentinel-5p (accessed March 30, 2020).

[15] Wu X Nethery RC Sabath MB Braun D Dominici F. Air pollution and COVID-19 mortality in the United States: strengths and limitations of an ecological regression analysis. Sci Adv. (2020) 6:eabd4049. 10.1126/sciadv.abd4049 33148655 PMC7673673

[16] Yao Y Pan J Liu Z Meng X Wang W Kan H. Ambient nitrogen dioxide pollution and spread ability of COVID-19 in Chinese cities. Ecotoxicol Environ Safety. (2020) 208:111421. 10.1101/2020.03.31.20048595 33038729 PMC7524685

[17] Liang D Shi L Zhao J Liu P Sarnat JA Gao S. Urban air pollution may enhance COVID-19 case-fatality and mortality rates in the United States. Innovation. (2020) 1:100047. 10.1016/j.xinn.2020.100047 32984861 PMC7505160

[18] Jiang Y Wu X-J Guan Y-J. Effect of ambient air pollutants and meteorological variables on COVID-19 incidence. Infect Control Hospital Epidemiol. (2020) 41:1–11. 10.1017/ice.2020.222 32389157 PMC7298083

[19] Setti L Passarini F De Gennaro G Barbieri P Perrone MG Borelli M. SARS-Cov-2 RNA found on particulate matter of bergamo in Northern Italy: first evidence. Environ Res. (2020) 188:109754. 10.1016/j.envres.2020.109754 32526492 PMC7260575

[20] Fattorini D Regoli F. Role of the chronic air pollution levels in the Covid-19 outbreak risk in Italy. Environ Pollut. (2020) 264:114732. 10.1016/j.envpol.2020.114732 32387671 PMC7198142

[21] Pluchino A Inturri G Rapisarda A Biondo AE Le Moli R Zappala' C A novel methodology for epidemic risk assessment: the case of COVID-19 outbreak in Italy. arXiv. 2004.02739 (2020).

[22] Setti L Passarini F De Gennaro G Barbieri P Licen S Perrone MG. Potential role of particulate matter in the spreading of COVID-19 in Northern Italy: first observational study based on initial epidemic diffusion. BMJ Open. (2020) 10:e039338. 10.1136/bmjopen-2020-039338 32973066 PMC7517216

[23] Pansini R Fornacca D Higher virulence of COVID-19 in the air-polluted regions of eight severely affected countries. medRxiv. (2020). 10.1101/2020.04.30.20086496

[24] Pansini R Fornacca D Initial evidence of higher morbidity and mortality due to SARS-CoV-2 in regions with lower air quality. medRxiv. (2020). 10.1101/2020.04.04.20053595

[25] Burge HA Biological airborne pollutants. In: Foster WM, Costa DL, editors. Lung Biology in Health Disease. Boca Raton, FL: Taylor & Francis Group (2005).

[26] Wong G Ko F Lau T Li S Hui D Pang S. Temporal relationship between air pollution and hospital admissions for asthmatic children in Hong Kong. Clin Exp Allergy. (2001) 31:565–9. 10.1046/j.1365-2222.2001.01063.x 11359423

[27] Smets W Moretti S Denys S Lebeer S Airborne bacteria in the atmosphere: presence, purpose, and potential. Atmos Environ. (2016) 139:214–21. 10.1016/j.atmosenv.2016.05.038

[28] Dong L Qi J Shao C Zhong X Gao D Cao W. Concentration and size distribution of total airborne microbes in hazy and foggy weather. Sci Total Environ. (2016) 541:1011–8. 10.1016/j.scitotenv.2015.10.001 26473703

[29] Wei K Zou Z Zheng Y Li J Shen F Wu C-y. Ambient bioaerosol particle dynamics observed during haze and sunny days in Beijing. Sci Total Environ. (2016) 550:751–9. 10.1016/j.scitotenv.2016.01.137 26849339

[30] Li Y Fu H Wang W Liu J Meng Q Wang W Characteristics of bacterial and fungal aerosols during the autumn haze days in Xi'an, China. Atmos Environ. (2015) 122:439–47. 10.1016/j.atmosenv.2015.09.070

[31] Cao C Jiang W Wang B Fang J Lang J Tian G. Inhalable microorganisms in Beijing's PM2.5 and PM10 pollutants during a severe smog event. Environ Sci Technol. (2014) 48:1499–507. 10.1021/es4048472 24456276 PMC3963435

[32] Ye Q Fu J-f Mao J-h Shang S-q. Haze is a risk factor contributing to the rapid spread of respiratory syncytial virus in children. Environ Sci Pollut Res. (2016) 23:20178–85. 10.1007/s11356-016-7228-6 27439752

[33] Chen P-S Tsai FT Lin CK Yang C-Y Chan C-C Young C-Y. Ambient influenza and avian influenza virus during dust storm days and background days. Environ Health Perspect. (2010) 118:1211–6. 10.1289/ehp.0901782 20435545 PMC2944079

[34] Chen G Zhang W Li S Williams G Liu C Morgan GG. Is short-term exposure to ambient fine particles associated with measles incidence in China? A multi-city study. Environ Res. (2017) 156:306–11. 10.1016/j.envres.2017.03.046 28388516

[35] Peng L Zhao X Tao Y Mi S Huang J Zhang Q. The effects of air pollution and meteorological factors on measles cases in Lanzhou, China. Environ Sci Pollut Res. (2020) 27:13524–33. 10.1007/s11356-020-07903-4 32030582

[36] Villeneuve Paul J Goldberg Mark S. Methodological considerations for epidemiological studies of air pollution and the SARS and COVID-19 coronavirus outbreaks. Environ Health Perspect. (2020) 128:095001. 10.1289/EHP7411 32902328 PMC7480171

[37] Haines A Ebi K. The imperative for climate action to protect health. N Engl J Med. (2019) 380:263–73. 10.1056/NEJMra1807873 30650330

