# Supplementary material for: COVID-19 Higher Mortality in Chinese Regions With Chronic Exposure to Lower Air Quality
Source: Front Public Health. 2021 Jan 22;8:597753. doi: 10.3389/fpubh.2020.597753 (PMC7874038; doi:10.3389/fpubh.2020.597753)
Supplement: Supplementary file 1 [file Data_Sheet_1.pdf]

COVID-19 higher morbidity and mortality in Chinese regions with lower air quality

Supplementary information

	unit	count	mean	std	min	25%	median	75%	max	range	iqr
**CO sat**	**μmol/m2**	347	40721	8751	15826	36202	42348	47034	60605	44778	10833
**NO_2 sat**	**μmol/m2**	347	57.58	42.62	5.00	26.00	42.70	83.85	185.39	180.42	57.85
**O_3 sat**	**μmol/m2**	347	130602	14073	115053	118774	126147	138924	170378	55325	20150
**SO_2 sat**	**μmol/m2**	347	58.61	65.11	-60.00	10.43	42.10	102.70	316.53	376.43	92.27
**Aerosol sat**	**index**	347	-0.97	0.17	-1.30	-1.06	-1.00	-0.92	-0.02	1.28	0.14
**HCHO sat**	**μmol/m2**	347	142.04	42.65	50.00	107.63	150.06	178.36	219.85	169.55	70.74
**PM 2.5 ground**	**AQI**	308	110.78	27.79	38.20	92.63	111.55	128.51	186.96	148.76	35.88
**PM 10 ground**	**AQI**	308	63.61	22.98	19.96	46.26	60.90	75.67	170.27	150.31	29.40
**CO ground**	**AQI**	308	9.71	3.97	2.39	7.11	8.74	11.85	27.01	24.63	4.74
**NO_2 ground**	**AQI**	308	13.72	5.40	3.17	9.63	13.40	17.41	28.57	25.40	7.78
**O_3 ground**	**AQI**	308	25.83	5.87	14.22	21.70	25.17	28.98	50.54	36.31	7.28
**SO_2 ground**	**AQI**	308	13.24	7.96	1.14	7.88	10.97	16.46	40.58	39.44	8.58

Table 1 S.I. Descriptive statistics for the different satellite- and ground-based air quality measurements. AQI = Air Quality Index, iqr = interquartile range. Negative values for the UV Aerosol Index (Aerosol sat) indicate that the amount of UV-absorbing aerosol detected by the sensor is inferior to a theoretical amount (see https://earth.esa.int/web/sentinel/technical-guides/sentinel-5p/level-2/aerosol-index, accessed on 31 October 2020). Negative values of SO_2 detected by satellite are due to noise in the data and are observed in particular over clean regions with low emissions (see https://developers.google.com/earth-engine/datasets/catalog/COPERNICUS_S5P_OFFL_L3_SO2, accessed on 31 October 2020).

	Population density		
	**df (n-2)**	**tau**	***p*-value**
**CO sat**	345	56	**<.001**
**NO_2 sat**	345	.65	**<.001**
**O_3 sat**	345	-.08	**.019**
**SO_2 sat**	345	.04	.246
**Aerosol sat**	345	-.11	**.003**
**HCHO sat**	345	.61	**<.001**
**PM 2.5 ground**	306	.34	**<.001**
**PM 10 ground**	306	.21	**<.001**
**CO ground**	306	.09	**.022**
**NO_2 ground**	306	.37	**<.001**
**O_3 ground**	306	.13	**.001**
**SO_2 ground**	306	.15	**<.001**

Table 2 S.I. Correlation between satellite- and ground-based air quality variables and population density in China.

China without Wuhan	Infections (/100k pop)			Fatalities (/100k pop)			Mortality (fatalities/infections)		
	df (n-2)	tau	*p*-value	df (n-2)	tau	*p*-value	df (n-2)	tau	*p*-value
CO sat	336	.28	**<.001**	336	.18	**<.001**	312	.16	**<.001**
NO_2 sat	336	.22	**<.001**	336	.14	**.001**	312	.12	**.008**
O_3 sat	336	-.08	**.031**	336	.00	.943	312	.02	.617
SO_2 sat	336	-.10	**.006**	336	-.02	.690	312	.00	.975
Aerosol sat	336	-.12	**.001**	336	-.03	.537	312	.00	.992
HCHO sat	336	.34	**<.001**	336	.19	**<.001**	312	.16	**<.001**
PM 2.5 ground	301	.15	**<.001**	301	.18	**<.001**	284	.17	**<.001**
PM 10 ground	301	.04	.361	301	.12	**.008**	284	.13	**.006**
CO ground	301	-.01	.798	301	.11	**.013**	284	.12	**.008**
NO_2 ground	301	.11	**.003**	301	.12	**.009**	284	.11	**.012**
O_3 ground	301	-.03	.512	301	-.02	.637	284	-.03	.528
SO_2 ground	301	-.01	.851	301	.04	.396	284	.06	.171
population	336	.22	**<.001**	336	.16	**<.001**	312	.14	**.002**
pop density	336	.32	**<.001**	336	.15	**<.001**	312	.12	**.007**

Table 3 S.I. Correlation between satellite- and ground-based air quality variables and cumulated COVID-19 infections per 100,000 inhabitants, fatalities per 100,000 inhabitants, and mortality rate in China (without the administrative unit of Wuhan), until 23 May 2020.

China without Hubei prov.	Infections (/100k pop)			Fatalities (/100k pop)			Mortality (fatalities/infections)		
	df (n-2)	tau	*p*-value	df (n-2)	tau	*p*-value	df (n-2)	tau	*p*-value
CO sat	320	.26	**<.001**	320	.13	**.002**	296	.11	**.011**
NO_2 sat	320	.26	**<.001**	320	.15	**.001**	296	.13	**.005**
O_3 sat	320	-.07	.054	320	.03	.551	296	.04	.417
SO_2 sat	320	-.08	**.044**	320	.04	.386	296	.05	.275
Aerosol sat	320	-.10	**.008**	320	.03	.539	296	.05	.299
HCHO sat	320	.32	**<.001**	320	.14	**.002**	296	.11	**.016**
PM 2.5 ground	286	.10	**.011**	286	.11	**.018**	269	.11	**.022**
PM 10 ground	286	.00	.992	286	.07	.108	269	.08	.087
CO ground	286	-.03	.395	286	.09	.052	269	.10	**.032**
NO_2 ground	286	.13	**.001**	286	.15	**.002**	269	.14	**.003**
O_3 ground	286	-.02	.668	286	-.01	.858	269	-.02	.697
SO_2 ground	286	-.01	.876	286	.04	.338	269	.06	.179
population	320	.26	**<.001**	320	.21	**<.001**	296	.19	**<.001**
pop density	320	.35	**<.001**	320	.19	**<.001**	296	.15	**<.001**

Table 4 S.I. Correlation between satellite- and ground-based air quality variables and cumulated COVID-19 infections per 100,000 inhabitants, fatalities per 100,000 inhabitants, and mortality rate in China (without Hubei province), until 23 May 2020.
